# Comparative safety profiles of baricitinib and tofacitinib in the treatment of adult alopecia areata: a pharmacovigilance study based on the FAERS database

**DOI:** 10.3389/fpubh.2025.1721315

**Published:** 2026-01-12

**Authors:** Lingsong Kong, Xu Liu, Yanhuan Feng

**Affiliations:** 1 Department of Operation and Logistics Management, West China Tianfu Hospital, Sichuan University, Chengdu, Sichuan, China; 2 Department of Dermatology, West China Hospital, Sichuan University, Chengdu, China; 3 Department of Nephrology, Institute of Kidney Diseases, West China Hospital of Sichuan University, Chengdu, China

**Keywords:** baricitinib, tofacitinib, adult alopecia areata, FAERS, pharmacovigilance

## Abstract

**Objective:**

To provide a comparative overview of the post-marketing safety patterns of baricitinib and tofacitinib used for the treatment of adult alopecia areata (AA) by analyzing spontaneous reports from the FDA Adverse Event Reporting System (FAERS). Given the different regulatory approval statuses and real-world usage contexts of the two drugs, this study aims to identify potential safety signals rather than to estimate incidence or establish causal associations.

**Methods:**

FAERS data from 2004Q1 to 2024Q1 were retrieved and systematically cleaned using FDA-recommended deduplication procedures. Reports involving baricitinib or tofacitinib with adult AA as the indication were included. Adverse events were standardized using MedDRA (v27.0). Disproportionality analyses were conducted using ROR, PRR, BCPNN, and MGPS. Subgroup analyses were performed by age, gender, and report severity. Statistical comparisons of categorical variables were conducted using χ^2^ or Fisher’s exact tests where appropriate. As FAERS lacks drug-exposure denominators, the results reflect reporting disproportionality rather than comparative risk.

**Results:**

A total of 550 baricitinib reports (941 events) and 648 tofacitinib reports (1927 events) were identified. The proportion of reports classified as serious was higher for baricitinib than for tofacitinib (*p* < 0.05), although this may be influenced by differences in approval status, reporting patterns, and market duration. For baricitinib, disproportionality signals were mainly observed in infections, general disorders, and laboratory investigations. For tofacitinib, signals were primarily related to injury, poisoning, and medication-related issues, consistent with its off-label use for AA. Subgroup analyses suggested variations in signal patterns across gender and age groups. No causal relationships can be inferred from these findings.

**Conclusion:**

Baricitinib and tofacitinib exhibit distinct post-marketing safety signal patterns when used in adult AA, likely influenced by their different regulatory indications and real-world utilization contexts. These pharmacovigilance results should be interpreted as hypothesis-generating and may help guide clinical vigilance and future analytical studies. Prospective research with controlled exposure data is needed to validate the safety signals identified in FAERS.

## Introduction

Alopecia areata (AA) is a chronic, relapsing autoimmune disorder characterized by non-scarring hair loss, substantially affecting patients’ psychological well-being and quality of life. Its pathogenesis involves complex interactions among genetic predisposition, autoimmune activation, and environmental triggers, yet remains incompletely understood (1–3). Although conventional therapies—such as corticosteroids, contact immunotherapy, and phototherapy—have been widely used, their efficacy is variable and long-term control remains challenging (4).

In recent years, Janus kinase (JAK) inhibitors have emerged as a major therapeutic advance in AA due to their targeted immunomodulatory effects on the JAK–STAT signaling pathway (5). Baricitinib, a selective JAK1/JAK2 inhibitor, became the first FDA-approved systemic treatment for severe AA in 2022, supported by robust phase 3 clinical trial data (6) and subsequent real-world evidence demonstrating sustained efficacy over 52 weeks (7). Tofacitinib, a JAK1/JAK3 inhibitor, has also shown clinical benefit in AA but is used exclusively off-label for this indication (8). Despite the growing use of JAK inhibitors in routine care, their comparative safety characteristics in real-world AA populations remain poorly defined.

Post-marketing pharmacovigilance offers complementary insight beyond clinical trials, especially for identifying rare or unexpected adverse events. The FDA Adverse Event Reporting System (FAERS) represents one of the largest spontaneous reporting databases worldwide and is widely used for signal detection through disproportionality analyses (9–11). However, its application to AA treatments requires careful interpretation, given the inherent limitations of voluntary reporting systems—including under-reporting, stimulated reporting, missing exposure denominators, and confounding from concomitant therapies (12, 13).

Importantly, baricitinib and tofacitinib differ substantially in regulatory approval status, usage contexts, and patient populations. These differences may influence the volume, type, and severity of reported adverse events. Therefore, FAERS cannot be used for head-to-head risk comparison or estimation of incidence; rather, it allows characterization of reporting patterns and identification of potential safety signals that warrant further evaluation. Against this background, the present study aims to systematically analyze FAERS data to characterize post-marketing adverse event signal patterns associated with baricitinib and tofacitinib when used in adult AA. By integrating multiple signal detection algorithms and conducting subgroup analyses by age, gender, and severity, this study seeks to provide hypothesis-generating evidence that may inform clinical vigilance and guide future analytical research.

## Methods

### Data source and study design

This retrospective pharmacovigilance study utilized data from the U.S. Food and Drug Administration Adverse Event Reporting System (FAERS) spanning from the first quarter of 2004 to the first quarter of 2024. FAERS is a large spontaneous reporting system designed to support post-marketing drug safety surveillance. Because the database relies on voluntary submissions and lacks information on the total number of drug-exposed patients, the findings can only reflect reporting disproportionality rather than true incidence or comparative risk. All quarterly datasets, including DEMO, DRUG, REAC, OUTC, THER, INDI, and RPSR files, were downloaded from the official FDA website and processed according to FDA recommendations and established methodological standards for FAERS-based signal detection.

### Case identification and eligibility criteria

Reports were included if baricitinib or tofacitinib was listed as a suspect drug (primary or secondary) and alopecia areata was identified as the therapeutic indication for adult patients. The study considered reports submitted within the defined time range and excluded pediatric cases. Reports with incomplete essential information—such as missing age, sex, adverse event onset date, or drug–event linkage—were removed to ensure data integrity. This approach allowed the construction of a clean dataset that ensured consistency in demographic and clinical characterization.

### Data cleaning and deduplication

A rigorous data-cleaning workflow was adopted to address the inherent issues of spontaneous reporting systems. Duplicate entries were identified and removed using the FDA-recommended CASEID–PRIMARYID hierarchy, retaining the most recent version when multiple submissions referred to the same case. Follow-up reports were merged in accordance with FDA guidelines to avoid inflating the event count. Drug names were standardized through MedDRA version 27.0 and the FDA Drug Dictionary, ensuring consistent identification of both suspect and concomitant medications. Implausible or inconsistent values, including negative onset intervals, were recoded as missing. Because concomitant medications may increase confounding, they were carefully reviewed, harmonized, and interpreted with caution during signal evaluation. A flowchart of the data cleaning process has been incorporated into the revised manuscript to enhance transparency and reproducibility.

### Adverse event coding and classification

All adverse events were coded according to Preferred Terms (PTs) and System Organ Classes (SOCs) under MedDRA version 27.0. Since individual FAERS reports may contain multiple PTs, each adverse event was treated as a separate event-level record rather than a patient-level outcome. This classification allowed a systematic description of the distribution of AEs and facilitated subsequent disproportionality analyses across organ systems.

### Statistical analysis

Descriptive statistics were generated for demographic and clinical characteristics. Categorical variables, such as the proportion of serious versus non-serious reports and the distribution of SOCs, were compared using χ^2^ tests. Fisher’s exact test was applied when expected cell counts were fewer than five. Continuous variables that did not follow a normal distribution, such as age or onset time, were summarized using medians and interquartile ranges and compared using Mann–Whitney U tests. All analyses were performed using R software (version 4.2.3), and a two-sided *p* value <0.05 was considered statistically significant.

### Disproportionality signal detection

To identify potential adverse event signals, four complementary disproportionality algorithms were applied: Reporting Odds Ratio (ROR), Proportional Reporting Ratio (PRR), Bayesian Confidence Propagation Neural Network (BCPNN), and Multi-Item Gamma Poisson Shrinker (MGPS). These methods represent both frequentist and Bayesian approaches and have been widely used in pharmacovigilance research. Each algorithm applied established thresholds to determine whether a signal was present. Because FAERS lacks denominator data and is subject to under-reporting and reporting biases, the identification of a disproportionate signal does not indicate causality or risk magnitude. All detected signals should therefore be regarded as hypothesis-generating and require further validation in controlled studies.

### Subgroup analyses

To explore potential heterogeneity in reporting patterns, subgroup analyses were conducted based on patient gender, age group (18–44 years, 45–64 years, and ≥65 years), and report severity as defined by FDA criteria. Heat maps were generated to illustrate variations in PT-level signals across these subgroups. These analyses aimed to provide exploratory insights into demographic differences in reported AEs, recognizing that such findings may reflect reporting behavior rather than biological variation.

### Ethical considerations

FAERS contains de-identified public data and does not involve direct patient contact or identifiable personal information. Ethical approval and informed consent were therefore not required for this study.

## Results

A total of 550 baricitinib reports and 648 tofacitinib reports met the inclusion criteria after data cleaning, corresponding to 941 and 1,927 individual adverse events, respectively. The demographic characteristics demonstrated noticeable differences between the two drugs. As shown in Table 1, patients receiving baricitinib had a median age of 42 years, whereas age data were more frequently missing among tofacitinib reports. Female patients constituted the majority in both groups. The source of reports differed substantially; a higher proportion of baricitinib submissions originated from consumers, whereas tofacitinib reports more frequently came from healthcare professionals. These variations likely reflect the distinct approval status and clinical use history of the two drugs rather than true differences in safety outcomes.

**Table 1:** Analysis of patients’ basic information.*

Indicator	Baricitinib	Tofacitinib
Gender	Number (%)	Number (%)
Female (%)	328 (59.64)	410 (63.27)
Male (%)	137 (24.91)	208 (32.10)
Not specified (%)	85 (15.45)	30 (4.63)
Age		
<18 (%)	9 (1.64)	143 (22.07)
18–44 (%)	147 (26.73)	259 (39.97)
45–64 (%)	88 (16.00)	146 (22.53)
65 ≤ (%)	21 (3.82)	36 (5.56)
Not specified (%)	285 (51.82)	64 (9.88)
Age (Quantitative)		
*N* (Missing)	265 (285)	584 (64)
Mean (SD)	42.22 (16.11)	34.45 (17.57)
Median (Q1, Q3)	42.00 (27.00, 55.00)	33.00 (18.00, 47.00)
Min, Max	13.00, 86.00	6.00, 89.00
Reporting year		
2014 (%)	-	2 (0.31)
2015 (%)	-	3 (0.46)
2016 (%)	-	13 (2.01)
2017 (%)	-	27 (4.17)
2018 (%)	-	49 (7.56)
2019 (%)	-	76 (11.73)
2020 (%)	-	77 (11.88)
2021 (%)	3 (0.55)	58 (8.95)
2022 (%)	62 (11.27)	165 (25.46)
2023 (%)	346 (62.91)	135 (20.83)
2024 (%)	139 (25.27)	43 (6.64)
Reporter		
Consumer (%)	382 (69.45)	169 (26.08)
Not specified (%)	96 (17.45)	4 (0.62)
Pharmacist (%)	48 (8.73)	54 (8.33)
Physician (%)	24 (4.36)	115 (17.75)
Other health-professional (%)	-	306 (47.22)
Continent of occurrence		
North America (%)	377 (68.55)	629 (97.07)
Not specified (%)	126 (22.91)	6 (0.93)
Asia (%)	26 (4.73)	5 (0.77)
Europe (%)	19 (3.45)	4 (0.62)
Oceania (%)	1 (0.18)	3 (0.46)
South America (%)	1 (0.18)	1 (0.15)
Country of Occurrence		
United States of America (%)	377 (68.55)	629 (97.07)
Japan (%)	12 (2.18)	4 (0.62)
Germany (%)	5 (0.91)	2 (0.31)
France (%)	4 (0.73)	2 (0.31)
Saudi Arabia (%)	4 (0.73)	1 (0.15)
Continent of reporting		
North America (%)	503 (91.45)	634 (97.84)
Asia (%)	26 (4.73)	6 (0.93)
Europe (%)	19 (3.45)	4 (0.62)
Oceania (%)	1 (0.18)	3 (0.46)
South America (%)	1 (0.18)	1 (0.15)
Reporting country		
United States of America (%)	503 (91.45)	634 (97.84)
Japan (%)	12 (2.18)	4 (0.62)
Germany (%)	5 (0.91)	2 (0.31)
France (%)	4 (0.73)	2 (0.31)
Saudi Arabia (%)	4 (0.73)	1 (0.15)
Serious reports		
Non-serious (%)	465 (84.55)	576 (88.89)
Serious (%)	85 (15.45)	72 (11.11)
Outcome		
Life-threatening (%)	1 (0.18)	3 (0.46)
Hospitalization - initial or prolonged (%)	15 (2.73)	11 (1.70)
Disability (%)	1 (0.18)	0 (0.00)
Death (%)	3 (0.55)	1 (0.15)
Congenital anomaly (%)	0 (0.00)	0 (0.00)
Required intervention to prevent permanent impairment/damage (%)	2 (0.36)	0 (0.00)
Other (%)	67 (12.18)	66 (10.19)
Time from drug administration to adverse - event onset (Days)		
0–30d (%)	17 (3.09)	44 (6.79)
31–60d (%)	6 (1.09)	1 (0.15)
61–90d (%)	4 (0.73)	0 (0.00)
91–120d (%)	1 (0.18)	3 (0.46)
121–150d (%)	2 (0.36)	0 (0.00)
151–180d (%)	3 (0.55)	0 (0.00)
181–360d (%)	9 (1.64)	1 (0.15)
360d < (%)	8 (1.45)	2 (0.31)
Missing or abnormal values (\ < 0) (%)	500 (90.91)	597 (92.13)
Time from drug administration to adverse - event onset (Days)		
*N* (Missing)	50 (500)	51 (597)
Mean (SD)	170.26 (249.80)	61.37 (261.74)
Median (Q1, Q3)	68.00 (9.00, 235.00)	0.00 (0.00, 0.00)
Min, Max	0.00, 1461.00	0.00, 1708.00
Weight (KG)		
*N* (Missing)	21 (529)	99 (549)
Mean (SD)	63.61 (15.33)	66.84 (18.25)
Median (Q1, Q3)	61.69 (54.00, 78.00)	67.00 (54.43, 77.00)
Min, Max	35.00, 97.50	26.70, 125.00

* This mining report only presents common basic information results. For more detailed content. The outcome is based on the patient dimension, not specific adverse events. A single patient may have multiple outcomes in the database, so the sum of outcomes may not be 100%. Serious reports are based on the patient dimension, not specific adverse events. In the FAERS database, reports with outcomes filled in are considered serious, while reports without outcomes are non-serious. The adverse event onset date collected by the database refers to the date when the patient first experienced an adverse event (no specific adverse event name is specified). When calculating the time from drug administration to adverse event onset (days), values less than 0 days are imputed as missing values before statistical analysis. For this type of data, more attention is generally paid to the median.

In terms of seriousness, baricitinib showed a higher proportion of reports classified as serious, and this difference reached statistical significance (Table 1). However, interpretation should be cautious, as FAERS does not provide data on the number of exposed patients, and reporting behavior may differ across products. Serious outcomes such as hospitalization, life-threatening events, or disability accounted for a small proportion of reports in both cohorts. Time-to-onset information was available for a minority of cases and showed wide variability, limiting its interpretability.

**Figure 1:**
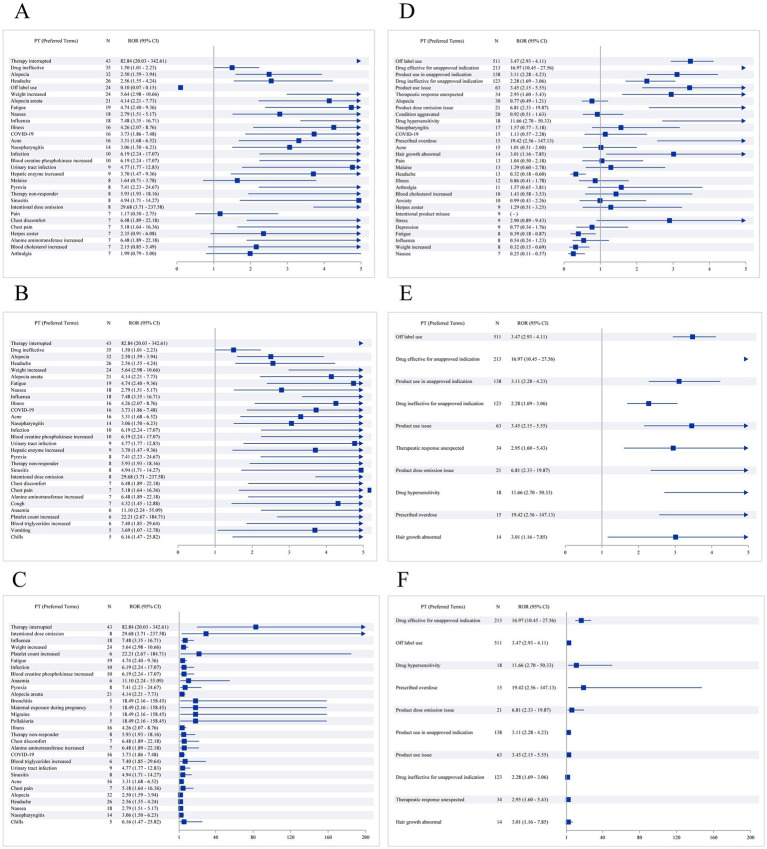
**(A)** Top 30 preferred terms of adverse reaction frequency for baricitinib; **(B)** Top 30 preferred terms of positive signal frequency for baricitinib; **(C)** Top 30 preferred terms of positive signal strength for baricitinib; **(D)** Top 30 preferred terms of adverse reaction frequency for tofacitinib; **(E)** Top 30 preferred terms of positive signal frequency for tofacitinib; **(F)** Top 30 preferred terms of positive signal strength for tofacitinib.

The distribution of adverse events across system organ classes (SOCs) differed between baricitinib and tofacitinib (Table 2). For baricitinib, the most frequently reported SOCs included general disorders and administration site conditions, infections and infestations, and investigations. In contrast, tofacitinib reports more often involved injury, poisoning, and procedural complications, followed by general disorders and skin-related events. These differences are further illustrated in the ranking of preferred terms (Figures 1A–F), where baricitinib reports more prominently featured systemic and infectious PTs, while tofacitinib was characterized by a higher frequency of medication-related or administration-related PTs. Such patterns should be interpreted in the context of differing clinical use environments and patient populations.

**Table 2:** Distribution of adverse events of the target drugs in different system organs.*

SOC	Events	Proportion (%)
Distribution of baricitinib adverse events in different system organs		
General disorders and administration site conditions	153	16.26
Infections and infestations	143	15.20
Investigations	118	12.54
Skin and subcutaneous tissue disorders	111	11.80
Nervous system disorders	65	6.91
Injury, poisoning and procedural complications	57	6.06
Gastrointestinal disorders	55	5.84
Surgical and medical procedures	54	5.74
Musculoskeletal and connective tissue disorders	34	3.61
Respiratory, thoracic and mediastinal disorders	32	3.40
Neoplasms benign, malignant and unspecified (incl cysts and polyps)	21	2.23
Vascular disorders	16	1.70
Psychiatric disorders	14	1.49
Blood and lymphatic system disorders	14	1.49
Cardiac disorders	11	1.17
Eye disorders	10	1.06
Renal and urinary disorders	9	0.96
Reproductive system and breast disorders	7	0.74
Metabolism and nutrition disorders	6	0.64
Immune system disorders	6	0.64
Hepatobiliary disorders	3	0.32
Ear and labyrinth disorders	1	0.11
Pregnancy, puerperium and perinatal conditions	1	0.11
Distribution of tofacitinib adverse events in different system organs		
Injury, poisoning and procedural complications	809	41.98
General disorders and administration site conditions	488	25.32
Skin and subcutaneous tissue disorders	123	6.38
Infections and infestations	89	4.62
Investigations	81	4.20
Psychiatric disorders	58	3.01
Nervous system disorders	46	2.39
Gastrointestinal disorders	41	2.13
Musculoskeletal and connective tissue disorders	35	1.82
Immune system disorders	34	1.76
Respiratory, thoracic and mediastinal disorders	32	1.66
Metabolism and nutrition disorders	12	0.62
Eye disorders	11	0.57
Blood and lymphatic system disorders	10	0.52
Neoplasms benign, malignant and unspecified (incl cysts and polyps)	9	0.47
Vascular disorders	9	0.47
Renal and urinary disorders	7	0.36
Surgical and medical procedures	6	0.31
Endocrine disorders	6	0.31
Hepatobiliary disorders	5	0.26
Social circumstances	5	0.26
Ear and labyrinth disorders	4	0.21
Cardiac disorders	3	0.16
Reproductive system and breast disorders	2	0.10
Product issues	1	0.05
Pregnancy, puerperium and perinatal conditions	1	0.05

* Events represent the number of adverse events occurring under the System Organ Class (SOC); Proportion = Number of adverse events occurring under the System Organ Class (SOC)/Total number of adverse events.

Disproportionality signal detection revealed several potential signals for each drug. For baricitinib, disproportionality appeared in infections, systemic reactions, and selected laboratory-related PTs. For tofacitinib, signals were concentrated in medication-use complications and injury-related categories. These observations emerged consistently across ROR, PRR, BCPNN, and MGPS methods, although Bayesian estimates such as EBGM tended to produce fewer signals, likely due to their conservative nature in smaller datasets. Importantly, all identified signals reflect patterns of reporting and cannot be interpreted as risk estimates or causal relationships.

**Figure 2:**
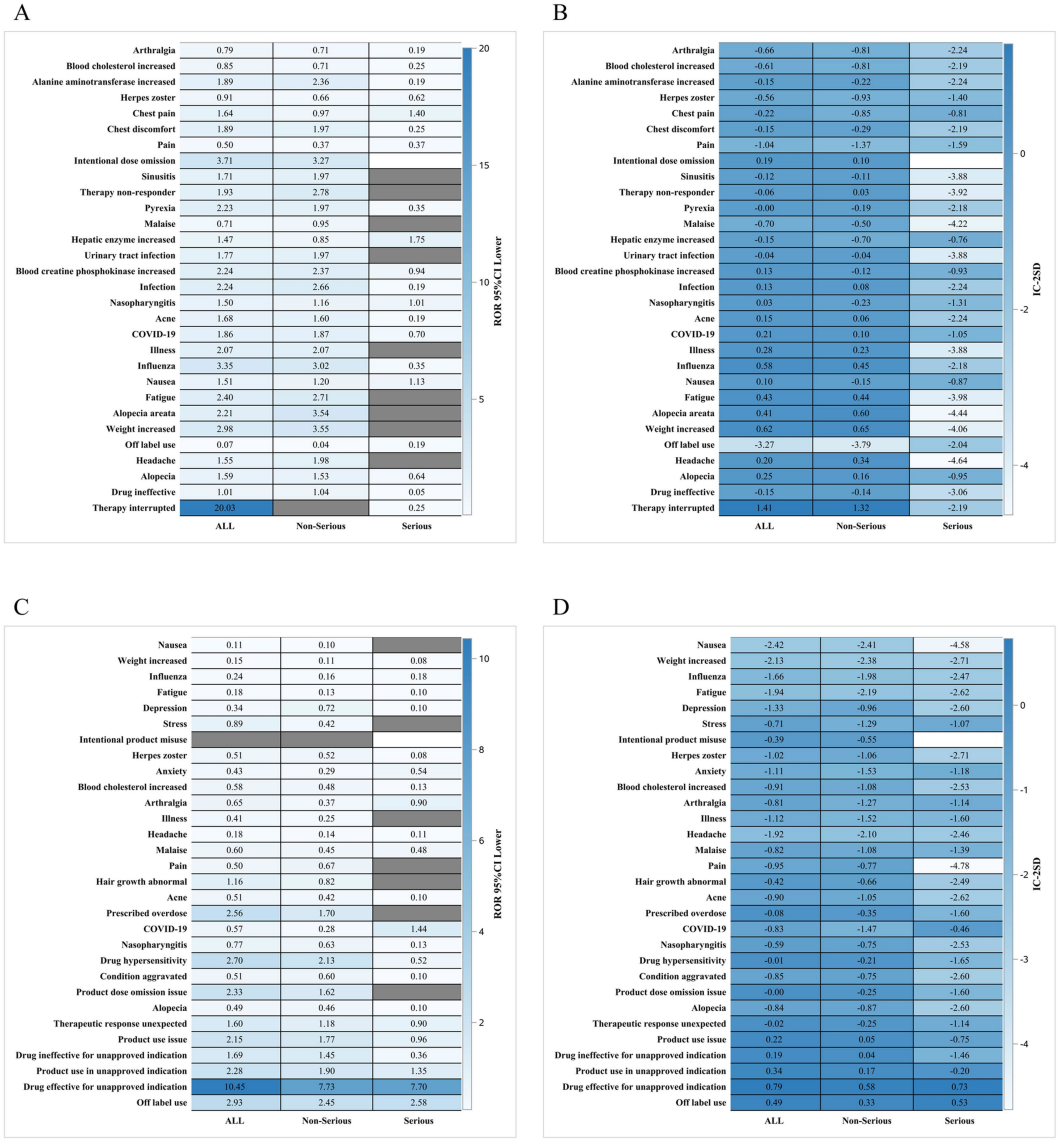
**(A,B)** Heat map of baricitinib data by gender subgroup; **(C,D)** Heat map of tofacitinib data by gender subgroup.

**Figure 3:**
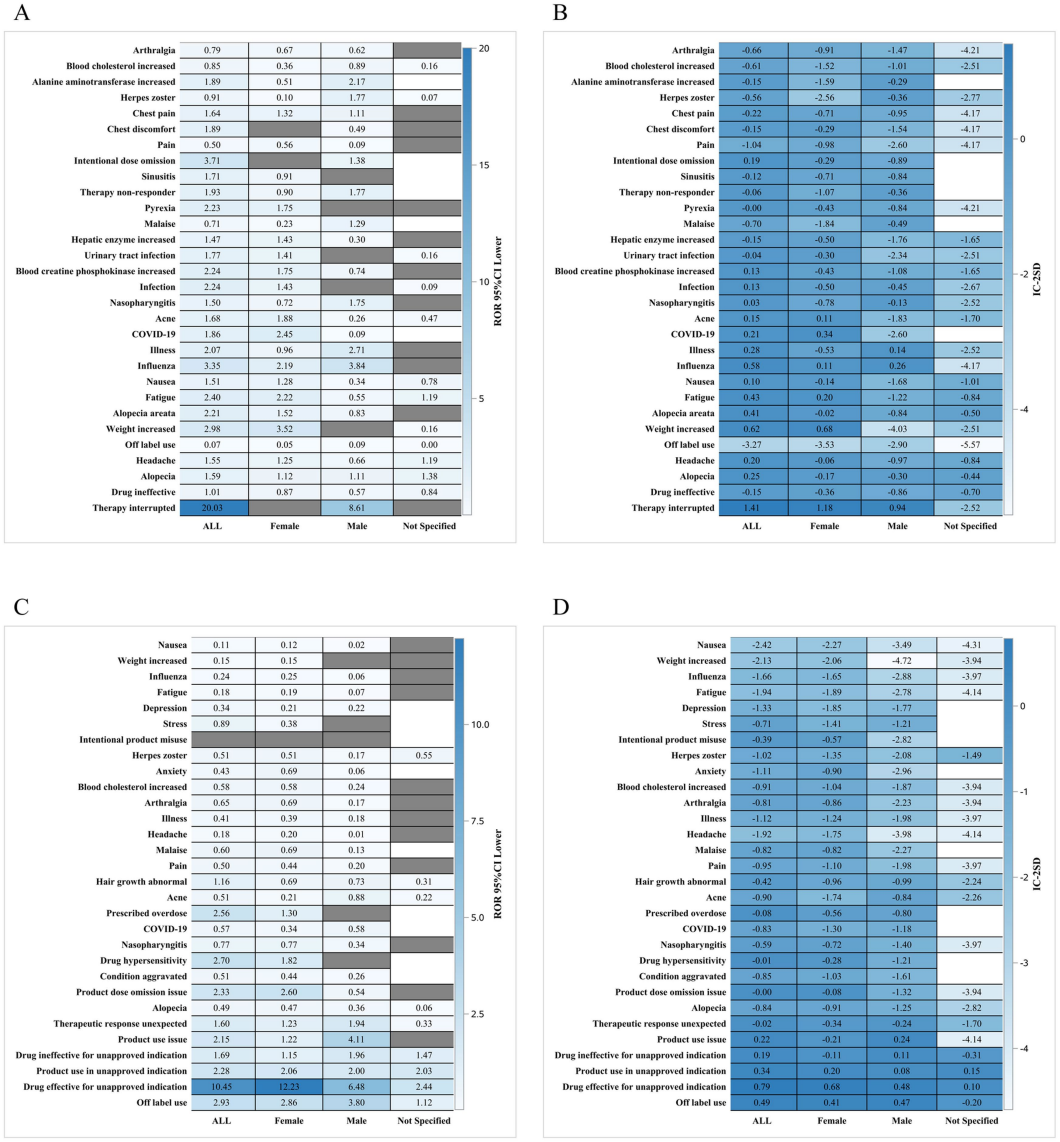
**(A,B)** Heat map of baricitinib data by age subgroup; **(C,D)** Heat map of tofacitinib data by age subgroup.

**Figure 4:**
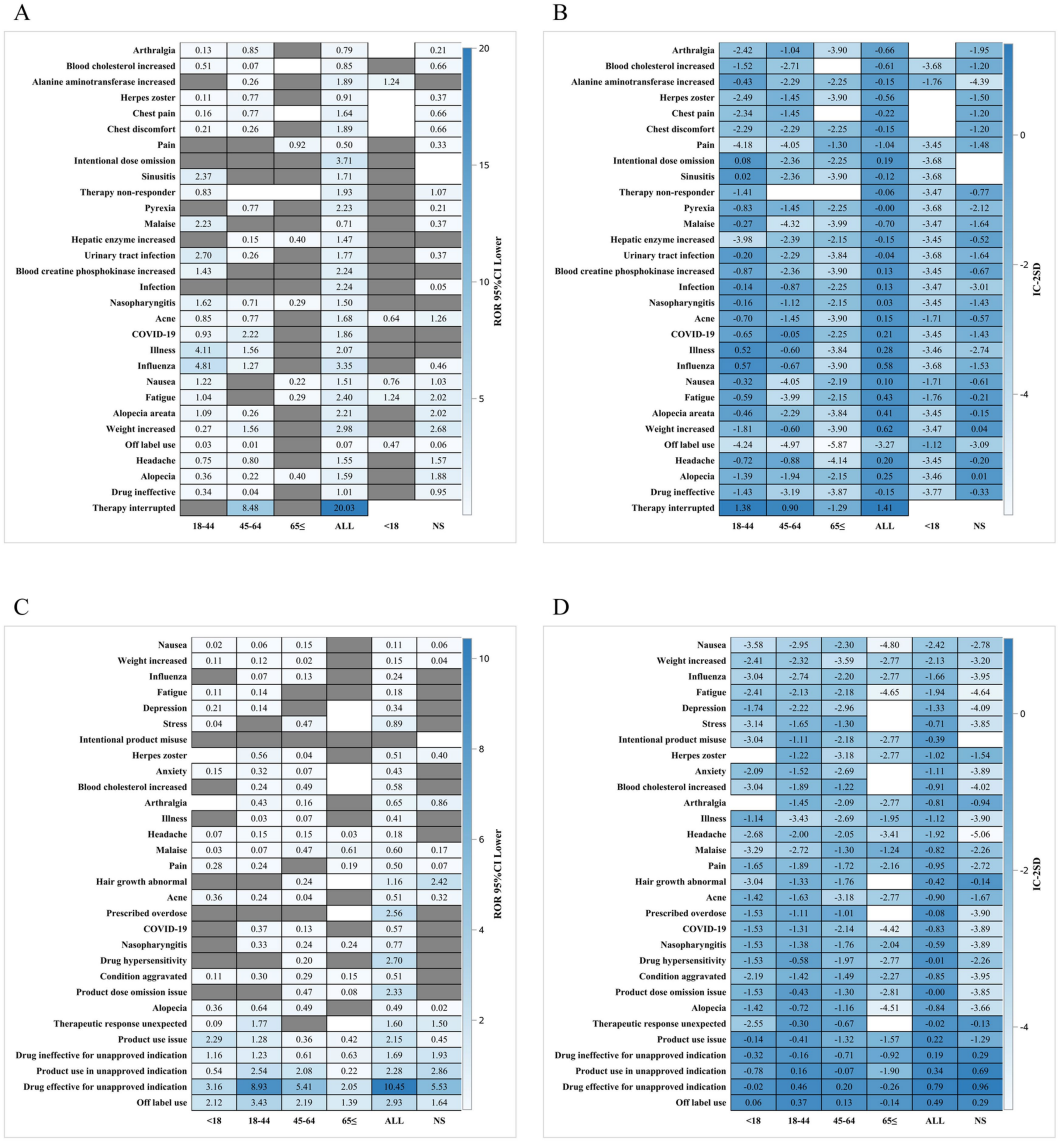
**(A,B)** Heat map of baricitinib data by serious report subgroup; **(C,D)** Heat map of tofacitinib data by serious report subgroup.

Subgroup analyses revealed meaningful variations in reporting patterns. The gender subgroup heatmap (Figures 2A–D) showed that male and female patients exhibited different distributions of adverse events, with female patients contributing more reports overall but males displaying a relatively higher proportion of systemic and neurologic terms. Age subgroup analysis (Figures 3A–D) demonstrated that older adults (≥65 years) showed a higher frequency of infection-related terms for baricitinib, while younger patients exhibited a more diverse PT distribution. Analysis of serious reports (Figures 4A–D) indicated that events categorized under vascular, neurologic, and general systemic disorders appeared more frequently in serious submissions for both drugs. These subgroup findings should be regarded as exploratory, as voluntary reporting may be influenced by access to care, drug availability, and reporting incentives.

Overall, the results demonstrate that baricitinib and tofacitinib display distinct patterns of adverse event reporting in FAERS. These patterns appear influenced not only by pharmacologic differences but also by their contrasting approval status, real-world usage contexts, and reporting behaviors. The observed disproportionality signals are hypothesis-generating and should serve as the basis for further validation in controlled studies.

## Discussion

This study provides a comprehensive pharmacovigilance assessment of post-marketing adverse event reporting patterns associated with baricitinib and tofacitinib when used for alopecia areata. Alopecia areata is a chronic autoimmune condition with complex immunologic and genetic underpinnings (1–3), and treatment historically relied on corticosteroids, contact immunotherapy, and other modalities with variable outcomes (4). The emergence of Janus kinase (JAK) inhibitors has transformed the therapeutic landscape (5), supported by mechanistic insights into the JAK–STAT pathway (5, 14). Baricitinib became the first FDA-approved systemic therapy for severe alopecia areata in 2022, with efficacy demonstrated in phase 3 clinical trials (6) and further validated in a 52-week multicenter real-world cohort study (7). Tofacitinib, while not FDA-approved for AA, has shown benefit in small open-label studies and is widely prescribed off-label (8). These divergent approval and usage contexts play a critical role in interpreting post-marketing safety data.

The present findings should be understood against this backdrop. Demographic and reporting differences between the two drugs were substantial, including age distribution, sex composition, and the type of reporters submitting FAERS entries (Table 1). Baricitinib reports more frequently originated from consumers, consistent with early adoption among patients and heightened awareness after its recent approval. Tofacitinib, by contrast, has been available for over a decade across multiple immune-mediated diseases, resulting in broader exposure and more clinician-submitted reports. Such structural differences likely contribute to variations in reporting frequency and severity classifications and limit the comparability of raw report counts.

Distinct patterns emerged in system organ class distributions (Table 2). Baricitinib-associated events were more frequently categorized under general disorders, infections, and laboratory investigations, consistent with known pharmacologic effects of JAK1/JAK2 inhibition and echoed by findings in both clinical trials and real-world studies (6, 7). Tofacitinib reports showed a higher proportion of injury, procedural complications, and medication-use–related events (Figure 1), a pattern that may reflect its longstanding multi-indication use and the complexity of patients receiving it. These findings align with prior literature documenting differences in adverse event profiles between JAK inhibitor subtypes (15, 16). Nonetheless, FAERS data do not allow causal interpretation or formal comparison of incidence.

Signal detection analyses further contextualized these patterns. Baricitinib demonstrated signals related to infections, systemic reactions, and abnormalities in laboratory parameters—biologically plausible effects given the drug’s mechanism. Tofacitinib showed signals involving medication administration, use complications, and injury categories. While ROR, PRR, BCPNN, and MGPS detected overlapping patterns, Bayesian estimates were more conservative, a known feature when case counts are limited (9–11). Across both drugs, signals represent disproportionality in reporting rather than evidence of increased risk and should be interpreted as hypothesis-generating.

Subgroup analyses added further nuance. Gender stratification revealed differential patterns in systemic and neurologic reporting (Figure 2), while age analyses indicated that older adults (≥65 years) receiving baricitinib showed proportionally more infection-related reports (Figure 3). Serious event subgroup analyses demonstrated prominence of vascular, systemic, and neurologic terms for both drugs (Figure 4). These findings are consistent with the broader literature on immunomodulatory therapies in autoimmune diseases (12, 15). However, FAERS cannot confirm whether differences arise from biological susceptibility, drug exposure conditions, comorbidities, or variations in reporting behavior.

Comparison with existing clinical data reinforces several observations. Baricitinib’s known safety concerns—including infections, laboratory abnormalities, and acneiform reactions—were observed in both pivotal trials (6) and in the real-world cohort study (7), corresponding with signals noted in this analysis. Tofacitinib’s AE profile, including upper respiratory infections and laboratory changes, has been documented in open-label AA studies (8) and in broader autoimmune disease cohorts. FAERS findings do not contradict these established profiles but extend their scope by identifying additional patterns worthy of investigation (17, 18).

The limitations inherent to FAERS profoundly shape the interpretation of these findings. Under-reporting, stimulated reporting, and incomplete clinical context are well-recognized challenges (13). The lack of exposure denominators precludes estimation of incidence or comparative risk. Off-label use, which is common for tofacitinib in AA, has been shown to increase the likelihood of adverse event reporting (12), further complicating interpretation. Differences in approval status, monitoring intensity, indication breadth, and comorbidity burden introduce unavoidable confounding. Thus, the signals identified in this study cannot be interpreted as causal associations and should serve solely as a foundation for further hypothesis-driven research. Despite these limitations, this analysis contributes valuable insights into the evolving safety landscape of JAK inhibitors for alopecia areata. By integrating large-scale post-marketing data with multiple signal detection methodologies, the study highlights adverse event domains that warrant focused clinical attention, including infections and laboratory abnormalities for baricitinib and medication-use complications for tofacitinib. As JAK inhibitors continue to expand within dermatologic therapeutics, the integration of pharmacovigilance findings, controlled trial data, and real-world evidence will be essential for refining long-term safety monitoring strategies.

## Conclusion

In this large FAERS-based pharmacovigilance analysis, baricitinib and tofacitinib demonstrated distinct patterns of post-marketing adverse event reporting when used for alopecia areata. These patterns appeared to reflect not only pharmacologic differences between JAK inhibitor subtypes but also meaningful variation in approval status, real-world prescribing environments, and reporting behaviors. Potential signals involving infections, systemic reactions, and laboratory abnormalities were observed for baricitinib, while tofacitinib signals were more commonly related to medication-use complications and injury-associated categories. Because FAERS lacks exposure denominators, contains heterogeneous clinical context, and is influenced by multiple reporting biases, these findings cannot be used to infer incidence or causality. Instead, they should be interpreted as hypothesis-generating indicators that help identify areas warranting closer clinical attention. As JAK inhibitors continue to expand within the therapeutic landscape of alopecia areata, integrating pharmacovigilance data with controlled trials and real-world evidence will be crucial for characterizing long-term safety. The present analysis underscores the importance of ongoing, exposure-controlled studies to validate the signals identified in spontaneous reporting systems and to support the development of more refined safety monitoring strategies for patients receiving systemic JAK inhibition.

## Data Availability

The original contributions presented in the study are included in the article/supplementary material, further inquiries can be directed to the corresponding author.
